# Simulation and design of folded perovskite x-ray detectors

**DOI:** 10.1038/s41598-019-41440-6

**Published:** 2019-03-26

**Authors:** Henning Mescher, Elias Hamann, Uli Lemmer

**Affiliations:** 1 Light Technology Institute, Karlsruhe Institute of Technology (KIT), 76131 Karlsruhe, Germany; 2 Institute of Microstructure Technology, Karlsruhe Institute of Technology (KIT), 76344 Eggenstein-Leopoldshafen, Germany; 3 Institute for Photon Science and Synchrotron Radiation, Karlsruhe Institute of Technology (KIT), 76344 Eggenstein-Leopoldshafen, Germany

## Abstract

A variety of medical, industrial, and scientific applications requires highly sensitive and cost-effective x-ray detectors for photon energies ranging from keV to MeV. Adapting the thickness of polycrystalline or single crystal conversion layers especially to high-energy applications increases the complexity of fabrication and potentially decreases the performance of conventional direct conversion x-ray detectors. To tackle the challenges with respect to the active layer thickness and to combine the superior performance of single crystal materials with the low-cost nature of polycrystalline conversion layers, we investigate thin film x-ray detector technologies based on a folded device architecture. Analytical models simulating the sensitivity and the detective quantum efficiency (DQE) are used to evaluate the performance of folded detectors based on polycrystalline organic-inorganic perovskite semiconductors in various layout configurations and for different photon energies. Simulations of folded perovskite devices show high sensitivities. The DQE analysis introduces additional noise related boundary conditions for the folding length. A comparison with conventional detectors based on state of the art conversion materials at different photon energies demonstrates the potential of the folded detector layout as simulated sensitivities are comparable to single crystal detectors.

## Introduction

X-ray detectors are of pivotal importance for a wide range of applications including medical diagnostics^1–3^, nondestructive testing (NDT)^4–9^, and scientific research^10,11^. Photon energies ranging from few tens of keV, e.g., in diagnostic mammography^1^ to MeV, e.g., in on-site nondestructive inspection^9^ require different device architectures to ensure an efficient conversion of the impinging radiation to a measurable signal. Currently, indirect conversion detectors based on a scintillator coupled to photodiodes are primarily used^1,12,13^. However, the indirect approach suffers from degraded spatial resolution resulting from optical crosstalk even if structured scintillators are used. In case of higher photon energies, this degradation becomes even more severe as thicker scintillators are required to ensure an efficient x-ray absorption.

**Figure 1:**
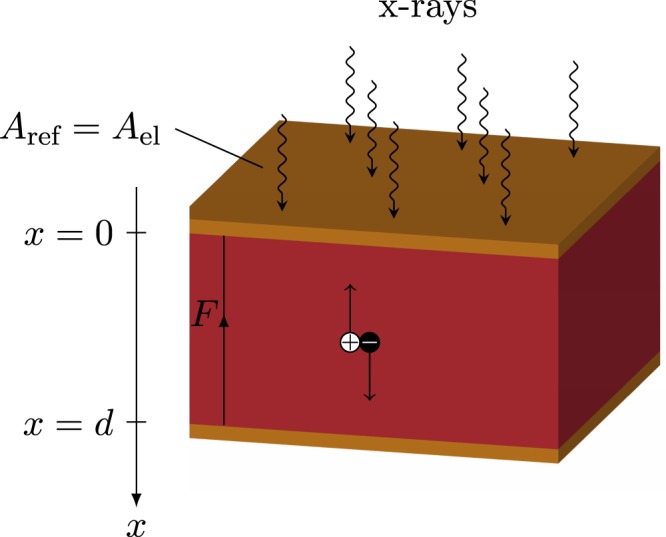
Scheme of device operation in a conventional device architecture for the case of a negatively biased top electrode. The red part represents the direct conversion material while the yellow parts indicate the electrodes.

In contrast, direct conversion detectors consist of a semiconducting material sandwiched between two electrodes enabling a direct conversion of absorbed photons to electric charges (see Fig. 1). Consequently, optical crosstalk within the absorbing layer is avoided improving the spatial resolution compared to indirect detectors and enabling an efficient x-ray absorption by adapting the active layer thickness. However, even in direct conversion detectors the spatial resolution is limited by charge sharing effects, e.g., due to an expansion of the charge carrier cloud or K-fluorescence re-absorption.

So far, amorphous selenium (a-Se) directly deposited on top of the readout electronics has the largest market share of direct conversion (photoconductive) detectors. However, its relatively low atomic number results in an insufficient absorption of higher energy radiation limiting its application to mammography^14^. As a result, the investigation of semiconducting materials that combine good x-ray absorption, high photon to charge conversion, and excellent charge transport properties is the subject of intense current research. Growing attention has been paid to hybrid pixel technologies^15^ where the sensor material and the read out electronics are processed on different substrates and electrically connected with micro-bumps afterwards. Consequently, single crystal semiconductors with sufficiently high atomic number such as CdZnTe^16^ can be utilized as detector material. However, in case of CdZnTe large differences in the charge transport of electrons and holes^17–20^ result in signals that are dependent on the depth of the x-ray interaction and energy resolved measurements require single polarity charge sensing with complex electrode structures^21^. A novel promising semiconductor material that has recently proven its ability to be used as x-ray conversion material and that can also be grown in high quality single crystals is hybrid organic-inorganic perovskite^22^. Detectors based on, e.g., single crystal methylammonium lead iodide (MAPbI_3_) have demonstrated high x-ray sensitivities^23^. However, among others, material degradation and ion migration in perovskite semiconductors remain issues to be solved. In general, the single crystal state of a direct conversion material provides optimal charge transport properties. Consequently, x-ray detectors based on single crystal semiconductors enable high sensitivity and high detective quantum efficiency (DQE). However, especially in the case of compound semiconductors, the fabrication of high quality single crystals is a complex and therefore expensive process and the crystal size is limited to several millimeters due to technological constraints.

As a consequence, and especially with respect to x-ray imaging detectors, large area processable polycrystalline semiconductors that can be directly processed to the read out electronics are of particular interest. A lot of attention has been paid to improve the properties of poly-CdZnTe. However, the most promising results are based on close-space sublimation deposition that utilizes high temperatures^24–26^. Such high temperatures are challenging especially for the direct implementation of the read out electronics. Conversion layers made of poly-PbI_2_ and poly-HgI_2_ enable highly sensitive x-ray detectors, but image lag and nonuniform pixel signals^27,28^ remain issues yet to be overcome. 1mm-thick poly-MAPbI_3_ layers have demonstrated very promising charge transport properties enabling the detection of higher photon energies more efficiently as compared to a-Se^29,30^. However, among others these approaches suffer from high dark current^29^ and degraded spatial resolution, potentially caused by charge sharing effects^30^. The feasibility of a quasi-direct conversion approach based on scintillator-sensitized hybrid organic active layers with thicknesses up to 170 *μ*m has been demonstrated, but small charge carrier drift-lengths^31^ limit the efficiency of such devices. Commonly, polycrystalline conversion materials enable large area processing at reduced costs but lower performance compared to their single crystal counterpart due to their non optimal charge transport properties. In addition, extremely large thicknesses of the active layer might even be impossible for polycrystalline semiconductors due to technological or temporal constraints, e.g., for vacuum deposition methods.

Whether single crystal or polycrystalline conversion materials are utilized, the thickness adaption of the active layers especially to higher photon energies is generally complex as high electric fields and high applied voltages are required to ensure an efficient charge extraction. This increases the noise due to high dark currents and moreover is a risk for the read out electronics. Furthermore, thicker active layers degrade both the spatial as well as the energy resolution by charge sharing effects, e.g., due to an expansion of the charge carrier cloud caused by diffusion and Coulomb repulsion or K-fluorescence re-absorption. Additionally, even in case of semiconductors with high quality charge transport properties, the finite lifetimes of the charge carriers make extremely thick active layers inefficient.

**Figure 2:**
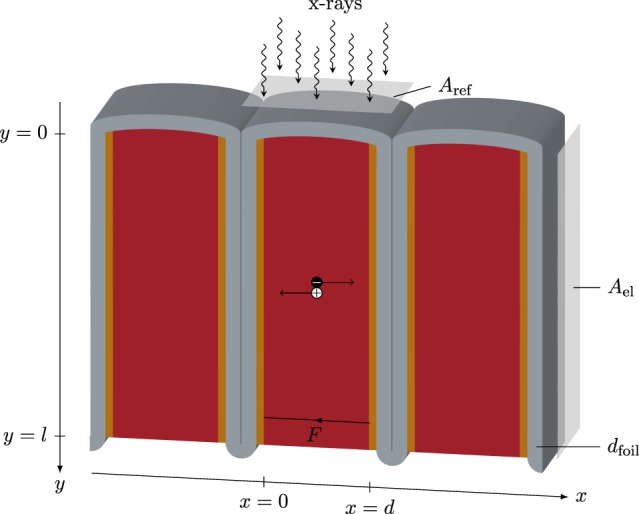
Scheme of device operation in a folded device architecture for the case of an one dimensional array. The red part represents the direct conversion material while the yellow parts indicate the electrodes. The flexible substrate is shown in grey. With the opening oriented to the +*y*-direction single folds can be connected to the readout electronics through the bottom of the array.

Motivated by the ability of polycrystalline perovskite semiconductors to be used as direct conversion materials in x-ray detectors^22,30^ in combination with their ability to be processed at low costs over large area and on flexible substrates, this work investigates the potential of *folded* (see Fig. 2) perovskite x-ray detectors. The folded architecture enables to decouple absorption and charge collection as x-rays are absorbed parallel to the collecting electrodes. Consequently, the proposed architecture can tackle the challenges with respect to the active layer thicknesses and can enable high x-ray absorption efficiency as the length of one fold can be optimized without affecting the charge collection. Furthermore, high detector performance can be provided even in case of materials with non optimal charge transport properties as the active layer thickness can be adapted without changing the x-ray absorption efficiency. Thus, the folded architecture has the potential to combine the optimal detector performance as in single crystals with the ability of polycrystalline conversion layers to be processed over large area at low costs. Edge-on detector geometries with the x-ray absorption parallel to the collecting electrodes^32–34^ bear some similarities to our approach and have been investigated with focus on specific aspects such as the count rate problem in spectral computed tomography (CT)^33,34^ and the application in positron emission tomography (PET)^32^. In this paper, we model and simulate the performance of folded perovskite x-ray detectors in order to deduce design rules for low-, mid-, and high-energy x-ray applications.

## Methods

We are aiming for an evaluation of the intrinsic performance of the folded detector design (see Fig. 2) compared to the conventional planar layout (see Fig. 1) with respect to sensitivities and detective quantum efficiencies. Consequently, within both the sensitivity model as well as within the model of the detective quantum efficiency no specific electrode structure is assumed and small pixel effects are neglected.

### Sensitivity

The sensitivity *S* of a photoconductive direct conversion x-ray detector is an important performance metric and is defined as the collected charge *Q* per unit exposure of radiation *X* per unit area *A*^35^:

1 $$S=\frac{Q}{XA}\,.$$

Following the approach in refs^35–38^, a uniform electric field *F* across a photoconductor with thickness *d* is considered. We assume the loss of charge carriers to be dominated by trap-assisted recombination and assign a constant mobility *μ* and a constant lifetime $\tau$ to each species of charge carriers. Under the assumption of two planar non pixelated electrodes the collected charge in the external circuit *Q* can be determined by integrating the induced currents described by the Shockley-Ramo theorem^21,39,40^ over the respective transit time:

2 $$Q=\frac{e{A}_{{\rm{el}}}F}{d}[{\mu }_{e}\,{\int }_{0}^{\frac{d}{{\mu }_{e}F}}\,{\int }_{0}^{d}\,n(x,t)\,dx\,dt+{\mu }_{h}\,{\int }_{0}^{\frac{d}{{\mu }_{h}F}}\,{\int }_{0}^{d}\,p(x,t)\,dx\,dt].$$

Here, *e* is the elementary charge and *A*_el_ is the area of electrodes. Neglecting furthermore the effect of charge carrier diffusion, the collected charge in the external circuit due to charge carrier drift can be modeled by analytically solving a simplified continuity equation for the electron and hole densities *n*(*x*, *t*) and *p*(*x*, *t*):

3 $${\partial }_{t}n(x,t)=\mp \,{\mu }_{e}F{\partial }_{x}n(x,t)-\frac{n(x,t)}{{\tau }_{e}},$$

4 $${\partial }_{t}p(x,t)=\pm \,{\mu }_{h}F{\partial }_{x}p(x,t)-\frac{p(x,t)}{{\tau }_{h}}.$$

Equations (2–4) assume initial electron and hole concentrations *n*(*x*, 0) and *p*(*x*, 0) created by a very short monoenergetic pulse of x-ray radiation. Comprehensive discussions of the underlying model assumptions can be found in refs^35,36^.

#### Conventional device architecture

For clarity, a brief outline of the analysis of conventional device architectures consisting of a planar top and bottom electrode (see Fig. 1) that is developed in refs^35,36^ is given before the model is extended to folded device architectures.

In case of the conventional device architecture (see Fig. 1), the solutions of Eqs (3) and (4) are given by drifting electron and hole distributions with an exponential shape and the collected charge modeled according to Eq. (2) can be expressed in terms of the x-ray absorption efficiency ${\eta }_{{\rm{x}}}$, the material dependent conversion efficiency ${\eta }_{{\rm{m}}}$, and the charge collection efficiency ${\eta }_{{\rm{cc}}}$

5 $$Q=e{A}_{{\rm{el}}}{\varphi }_{0}{\eta }_{{\rm{x}}}{\eta }_{{\rm{m}}}{\eta }_{{\rm{cc}}},$$

with

6 $${\eta }_{{\rm{x}}}=1-{e}^{-\alpha d},$$

7 $${\eta }_{{\rm{m}}}=\frac{E}{{W}_{\pm }}(\frac{{\alpha }_{{\rm{en}}}}{\alpha }),$$

8 $${\eta }_{{\rm{cc}}}={x}_{e}\,[1+\frac{{e}^{-1/{x}_{e}}-{e}^{-1/{\rm{\Delta }}}}{(1-{e}^{-1/{\rm{\Delta }}})({\rm{\Delta }}/{x}_{e}-1)}]+{x}_{h}\,[1-\frac{1-{e}^{-1/{\rm{\Delta }}-1/{x}_{h}}}{(1-{e}^{-1/{\rm{\Delta }}})({\rm{\Delta }}/{x}_{h}+1)}].$$

Here, ${\varphi }_{0}$ is the x-ray photon fluence, ${\rm{\Delta }}=1/(\alpha d)$ is the normalized attenuation depth with the attenuation coefficient *α*, *E* is the x-ray energy, *W*_±_ is the electron hole pair creation energy, *α*_en_ is the energy absorption coefficient and ${x}_{e}={\mu }_{e}F{\tau }_{e}/d$ and ${x}_{h}={\mu }_{h}F{\tau }_{h}/d$ are the normalized charge carrier schubwegs. In Eq. (8) we assume a negatively biased top electrode.

Considering furthermore the relation ${\varphi }_{0}=5.45\times {10}^{13}X/(E{({\alpha }_{{\rm{en}}}/\rho )}_{{\rm{air}}})$ between the x-ray photon fluence ${\varphi }_{0}$, the radiation exposure *X* and the mass energy absorption coefficient for air ${({\alpha }_{{\rm{en}}}/\rho )}_{{\rm{air}}}$^35,41^ in combination with Eq. (1), the sensitivity can be expressed as

9 $$S={S}_{0}{\eta }_{{\rm{x}}}{\eta }_{{\rm{m}}}{\eta }_{{\rm{cc}}},$$

with ${S}_{0}=5.45\times {10}^{13}e/(E{({\alpha }_{{\rm{en}}}/\rho )}_{{\rm{air}}})$. If *e* is in C, *E* in eV, and ${({\alpha }_{{\rm{en}}}/\rho )}_{{\rm{air}}}$ in cm^2^/*g*, then *S*_0_ is in C/(Rcm^2^)^35^. Assuming charged particle equilibrium, *S*_0_ can be converted in SI units C/(Gy_air_cm^2^) by multiplication with ${f}_{\text{conv}}={(8.76\times {10}^{-3}{{\rm{G}}{\rm{y}}}_{{\rm{a}}{\rm{i}}{\rm{r}}}/{\rm{R}})}^{-1}$ ^42^. Equation (9) further assumes an effective fill factor of ${\eta }_{{\rm{f}}}=1$ implying that the area of the electrodes *A*_el_ equals the reference area *A*_ref_ (see Fig. 1).

#### Folded device architecture

Different to the conventional architecture and under the assumption of an ideal parallel impinging beam, the x-ray absorption in case of a folded device is in parallel to the electrodes along the folding length *l* (see Fig. 2). Correspondingly, the initial electron and hole distributions are laterally homogeneous and are then driven out by the applied electric field. The collected charge according to Eq. (2) is

10 $$Q=e{A}_{{\rm{el}}}\frac{d}{l}{\varphi }_{0}{\eta }_{{\rm{x}},{\rm{F}}}{\eta }_{{\rm{m}}}{\eta }_{{\rm{cc}},{\rm{F}}},$$

with

11 $${\eta }_{{\rm{x}},{\rm{F}}}=1-{e}^{-\alpha l},$$

12 $${\eta }_{{\rm{cc}},{\rm{F}}}={x}_{e}^{2}\,({e}^{-1/{x}_{e}}-1)+{x}_{e}+{x}_{h}^{2}\,({e}^{-1/{x}_{h}}-1)+{x}_{h}.$$

Taking into consideration that for the folded architecture the area of the electrodes scales with the folding length (${A}_{{\rm{el}}}\propto l$, see Fig. 2) and the reference area with the total thickness of one fold (${A}_{{\rm{ref}}}\propto d+2{d}_{{\rm{foil}}}$, see Fig. 2) the sensitivity can be expressed as

13 $$S={S}_{0}{\eta }_{{\rm{f}}}{\eta }_{{\rm{x}},{\rm{F}}}{\eta }_{{\rm{m}}}{\eta }_{{\rm{cc}},{\rm{F}}},$$

with the effective fill factor

14 $${\eta }_{{\rm{f}}}=\frac{d}{d+2{d}_{{\rm{foil}}}}.$$

Here, the projected area of the electrodes (typically thinner than 150 nm) is neglected.

### Detective Quantum Efficiency

The detective quantum efficiency (DQE) is a further important performance metric measuring the signal and noise propagation in a detector and can be defined as^43^:

15 $${\rm{DQE}}=\frac{{{\rm{SNR}}}_{{\rm{out}}}^{{\rm{2}}}}{{{\rm{SNR}}}_{{\rm{in}}}^{{\rm{2}}}},$$

where SNR_in_ and SNR_out_ are the signal-to-noise ratios (SNR) at the input and the output stage of the detector. Following the approaches developed in refs^37,44–48^ a cascaded linear system model is used to analyze the DQE. Similar to investigations in refs^46,47^ spatial correlations are neglected and the DQE is analyzed at zero spatial frequency referred to as DQE(0).

**Figure 3:**
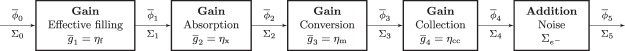
Block diagram of the applied linear system model illustrating the propagation of the signal ${\overline{\varphi }}_{i}$ and noise power ${{\rm{\Sigma }}}_{i}$ through the five stages. Stages (1–4) are modeled as stochastic amplification stage with mean gain ${\overline{g}}_{i}$, whereas stage (5) represents the addition of electronic noise ${{\rm{\Sigma }}}_{{e}^{-}}$.

According to the signal propagation developed within the sensitivity analysis, the applied linear system model shown in Fig. 3 consists of five stages: (1) effective filling, (2) x-ray absorption, (3) conversion to charge carriers, (4) charge collection, and (5) the addition of electronic noise. Each of the first four stages can be modeled as a stochastic amplification stage where the mean quantum fluence ${\overline{\varphi }}_{i}$ and noise power ${{\rm{\Sigma }}}_{i}$ at the stage *i* can be modeled as^44,49^:

16 $${\overline{\varphi }}_{i}={\overline{\varphi }}_{i-1}\,{\overline{g}}_{i},\,{{\rm{\Sigma }}}_{i}={\overline{g}}_{i}^{2}{{\rm{\Sigma }}}_{i-1}+{\sigma }_{{g}_{i}}^{2}{\overline{\varphi }}_{i-1},$$

with the mean gain ${\overline{g}}_{i}$ and variance of the gain ${\sigma }_{{g}_{i}}^{2}$. Assuming a binomial selection process in stage (1), (2), and (4) the variances can be calculated as^49^:

17 $${\sigma }_{{g}_{i}}^{2}={\overline{g}}_{i}(1-{\overline{g}}_{i})\,{\rm{for}}\,i=1,2,4.$$

Neglecting effects of K-fluorescence re-absorption and assuming the mean number of free electron hole pairs to be Poisson distributed, the variance of stage (3) is ${\sigma }_{{g}_{3}}^{2}={\overline{g}}_{3}$^37,47^. Stage (5) represents the addition of electronic noise ${{\rm{\Sigma }}}_{{e}^{-}}$ where the mean output signal and the total noise power can be modeled as^47^:

18 $${\bar{\varphi }}_{5}={\bar{\varphi }}_{4},\,{{\rm{\Sigma }}}_{5}={{\rm{\Sigma }}}_{4}+{{\rm{\Sigma }}}_{{e}^{-}}.$$

Assuming a Poisson distributed mean x-ray fluence ${\overline{\varphi }}_{0}$ impinging on the detector, the input noise power is ${{\rm{\Sigma }}}_{0}={\overline{\varphi }}_{0}$ and the DQE(0) can be modeled as:

19 $${\rm{DQE}}(0)=\frac{{\overline{\varphi }}_{5}^{2}}{{{\rm{\Sigma }}}_{5}}\frac{{{\rm{\Sigma }}}_{0}}{{\overline{\varphi }}_{0}^{2}}=\frac{{\eta }_{{\rm{f}}}{\eta }_{{\rm{x}}}{\eta }_{{\rm{m}}}{\eta }_{{\rm{cc}}}}{1+{\eta }_{{\rm{m}}}{\eta }_{{\rm{cc}}}+(\frac{{{\rm{\Sigma }}}_{{e}^{-}}}{{\overline{\varphi }}_{0}})\frac{1}{{\eta }_{{\rm{f}}}{\eta }_{{\rm{x}}}{\eta }_{{\rm{m}}}{\eta }_{{\rm{cc}}}}}.$$

In case of folded device architectures, the x-ray absorption efficiency is enhanced to the disadvantage of an increased total dark current per unit area *I*_d_/*A*_ref_. For this reason, the analysis of added electronic noise in stage (5) focuses on the contribution from dark current. Fluctuations in the dark current can be caused by shot noise as well as by 1/*f* noise. Investigations on the effect of 1/*f* noise in ref.^50^ show that 1/*f* contributions can be significant. However, the consideration of 1/*f* noise is beyond the scope of this study and 1/*f* contributions to the noise fluctuations are neglected at this stage. Thus, assuming the fluctuations in the dark current to be shot noise dominated, the dark current contribution to the noise power can be modeled as^14^:

20 $${{\rm{\Sigma }}}_{{e}^{-}}=\frac{{I}_{{\rm{d}}}{t}_{{\rm{int}}}}{{A}_{{\rm{ref}}}e},$$

where *t*_int_ is the integration time. Utilizing ${\overline{\varphi }}_{0}={\rm{\Psi }}{t}_{{\rm{int}}}$ with the incoming photon flux ${\rm{\Psi }}$, the dark current related part of Eq. (19) can be written as:

21 $$\frac{{{\rm{\Sigma }}}_{{e}^{-}}}{{\overline{\varphi }}_{0}}=\frac{{I}_{{\rm{d}}}}{{A}_{{\rm{ref}}}e{\rm{\Psi }}}.$$

Consequently, by comparing the number of electronic noise quanta due to dark current with the number of incoming photons both per unit time and unit area, the influence of the electronic noise can be analyzed independently from the integration time *t*_int_. For simplicity, a material dependent constant dark current density per unit electrode area *J*_d_ = *I*_d_/*A*_el_ is assumed. Accordingly, the total dark current per unit reference area in case of folded device architectures is:

22 $${(\frac{{I}_{{\rm{d}}}}{{A}_{{\rm{ref}}}})}_{{\rm{F}}}={J}_{{\rm{d}}}\frac{{A}_{{\rm{el}}}}{{A}_{{\rm{ref}}}}={J}_{{\rm{d}}}\frac{l}{d+2{d}_{{\rm{foil}}}}.$$

Note, that in the folded architecture *I*_d_/*A*_ref_ scales with *l*/$(d+2{d}_{{\rm{foil}}})$ as ${A}_{{\rm{el}}}\propto l$ and ${A}_{{\rm{ref}}}\propto d+2{d}_{{\rm{foil}}}$. We here note that the readout electronics utilized in combination with the folded detector design will generate additional electronic noise. However, the associated electronic noise is strongly dependent on the specific readout technology (e.g. thermal and amplifier noise in thin film transistor readout electronics^47^) and is therefore not part of the noise propagation analysis utilized in this study.

### Simulation parameters

Inspired by typical thin film devices and our printing capabilities, we have restricted the parameter variations in our simulations to active layers with a thickness $d\in [0.01\,\mu {\rm{m}},100\,\mu {\rm{m}}]$, and folding lengths $l\in [0.01\,{\rm{mm}},1000\,{\rm{mm}}]$. All simulations of folded device architectures assume a substrate foil with thickness ${d}_{{\rm{foil}}}=1.4\,\mu {\rm{m}}$ (cf. ref.^51^).

Unless otherwise stated, the electric field is $F=0.25\,{\rm{V}}/\mu {\rm{m}}$. Simulations are further based on three photon energies (a) $E=20\,{\rm{keV}}$, (b) $E=60\,{\rm{keV}}$, and (c) $E=500\,{\rm{keV}}$ approximately representing the various photon energies utilized in applications such as (a) mammography, x-ray diffraction, agricultural and food quality evaluation (b) chest radiography, luggage inspection, clinical computed tomography (CT), and (c) on-site NDT, cargo inspection and positron emission tomography (PET).

The investigated direct conversion materials are a-Se, polycrystalline PbI_2_, HgI_2_, CdZnTe, MAPbI_3_, and single crystal CdZnTe, and MAPbI_3_. The material parameters utilized in the simulations are summarized in Table 1. For MAPbI_3_ ambipolar charge transport with equal electron and hole mobilities ${\mu }_{e}\approx {\mu }_{h}$ is assumed^52–54^. This is in good agreement with electron and hole mobilities determined experimentally^22,30,55,56^. We further assume equal electron and hole lifetimes ${\tau }_{e}\approx {\tau }_{h}$. Measurements of the mobility and the mobility lifetime product for electrons and holes in single crystal MAPbI_3_ support this assumption^55^. Simulations of folded perovskite x-ray detectors are based on poly-MAPbI_3_ and utilize two material parameter configurations $\mu {\tau }_{{\rm{high}}}={10}^{-4}\,{{\rm{cm}}}^{2}/{\rm{V}}$ and $\mu {\tau }_{{\rm{low}}}={10}^{-6}\,{{\rm{cm}}}^{2}/{\rm{V}}$. $\mu {\tau }_{{\rm{high}}}$ is in good agreement with recently reported mobility lifetime measurements of poly-MAPbI_3_ used in a x-ray detector^30^. However, to account for the complexity of fabricating high-quality poly-MAPbI_3_ layers a low quality configuration $\mu {\tau }_{{\rm{low}}}$ is also considered.

**Table 1:** Material parameters used in the simulations with density $\rho$, electron hole pair creation energy *W*_±_, charge carrier mobility *μ*, charge carrier lifetime $\tau$, and electron (hole) mobility lifetime products ${\mu }_{e,(h)}{\tau }_{e,(h)}$.

	*ρ*[g/cm^3^]	*W*_±_[eV]	*μ*_*e*_*τ*_*e*_[cm^2^/V]
			*μ*_*h*_*τ*_*h*_[cm^2^/V]
a-Se	4.3^63^	45^63,64^	5.0 × 10^−7^ ^65,66^ 1.0 × 10^−6^ ^65,66^
poly-PbI_2_	6.0^63^	5^63^	7.0 × 10^−8^ ^63^ 2.0 × 10^−6^ ^63^
poly-HgI_2_	6.3^63^	5^63^	2.0 × 10^−5^ ^63,67^ 6.0 × 10^−6^ ^63,67^
poly-CdZnTe	5.8^63^	5^63^	2.0 × 10^−4^–2.4 × 10^−4^ ^14,17^ 4.0 × 10^−7^–3.0 × 10^−6^ ^14,17,68^
single-CdZnTe	5.8^63^	5^63^	1.0 × 10^−4^–1.0 × 10^−2^ ^17–20,69,70^ 4.0 × 10^−6^–1.0 × 10^−4^ ^17–20^
poly-MAPbI_3_ *μ* = 6.00 − 139 cm^2^/Vs^22,30,72–74^ *τ* = 0.01 − 8.00 *μ*s^30,74–76^	4.3^71^	5^22,38^	6.0 × 10^−8^–1.1 × 10^−3^
single-MAPbI_3_ *μ* = 2.50 − 164 cm^2^/Vs^52,53,55,56^ *τ* = 0.50 − 234 *μ*s^52,53,56^	4.3^71^	5^22,38^	1.3 × 10^−6^–3.8 × 10^−2^

In case of MAPbI_3_ mobility lifetime products $\mu \tau$ are calculated from reported mobilities *μ* and charge carrier lifetimes $\tau$. a-Se refers to stabilized amorphous selenium whereas poly-crystallinity (poly-) and single-crystallinity (single-) is indicated for all other active materials. Simulations of a-Se and PbI_2_ are based on a positively biased top electrode whereas HgI_2_ and CdZnTe utilize a negatively biased top electrode. The value of *W*_±_ for a-Se is at *F* = 10 V/*μ*m.

In order to conduct a realistic comparison with the optimal detector performance in the traditional architecture, simulations of the conventional planar layout utilize the maximum reported mobility lifetime products stated in Table 1 (Simulations of the conventional layout based on poly-MAPbI_3_ utilize $\mu {\tau }_{{\rm{high}}}={10}^{-4}\,{{\rm{cm}}}^{2}/{\rm{V}}$). Mass attenuation coefficients $\alpha /\rho$ and mass energy absorption coefficients ${\alpha }_{{\rm{en}}}/\rho$ are obtained from the National Institute of Standards and Technology data base^57^ where compound coefficients are based on the atomic mass weighted average of the elemental coefficients.

Simulations of the detective quantum efficiency consider two incoming photon fluxes. Firstly, we use ${\rm{\Psi }}={10}^{8}\,{\rm{1}}/({{\rm{mm}}}^{2}{\rm{s}})$ approximately representing the typical count rates in the unattenuated beam in clinical mammography, radiography, and CT^1,2^ (note, that count rates in non medical applications not focusing on the reduction of patient dose utilize even higher photon fluxes^7,58^, e.g. at synchrotron sources). Secondly we simulate a low flux case assuming a transmitted flux of ${{\rm{\Psi }}}_{{\rm{L}}}={10}^{-3}\times {\rm{\Psi }}{\mathrm{=10}}^{5}\,\mathrm{1/(}{{\rm{mm}}}^{2}{\rm{s}})$ in order to investigate the DQE(0) in an attenuated beam as well.

## Results and Discussion

### Layout optimization with respect to the sensitivity and the detective quantum efficiency

**Figure 4:**
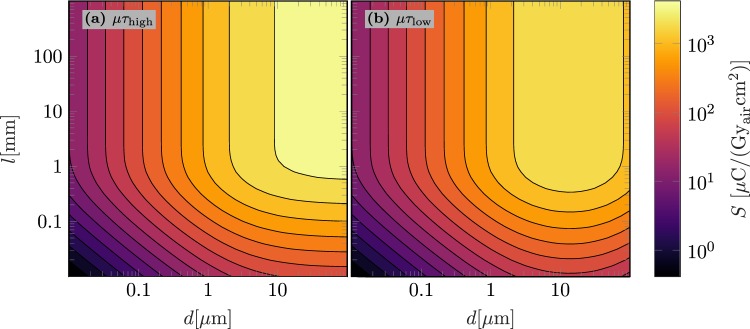
Simulated sensitivities *S* of folded poly-MAPbI_3_ x-ray detectors as a function of the active layer thickness *d* and the folding length *l*. (**a**) Use $\mu {\tau }_{{\rm{high}}}$ and (**b**) use $\mu {\tau }_{{\rm{low}}}$ to simulate *S* in the high and the low quality configuration. The x-ray energy is $E=60\,{\rm{keV}}$.

In order to evaluate the performance of poly-MAPbI_3_ in folded device architectures, Fig. 4 illustrates the simulated sensitivity *S* for different layout configurations (*l*, *d*) in the high and in the low quality configuration ($\mu {\tau }_{{\rm{high}},{\rm{low}}}$), exemplary at a photon energy of $E=60\,{\rm{keV}}$.

The layout related influences on *S* in Eq. (13) are the effective fill factor ${\eta }_{{\rm{f}}}$, the x-ray absorption ${\eta }_{{\rm{x}},{\rm{F}}}$, and the charge collection efficiency ${\eta }_{{\rm{cc}},{\rm{F}}}$. According to Eq. (11), ${\eta }_{{\rm{x}},{\rm{F}}}$ can be improved by increasing the absorption length *l*. According to Eq. (14), ${\eta }_{{\rm{f}}}$ can be improved by increasing the active layer thickness *d*. In contrast and according to Eq. (12), ${\eta }_{{\rm{cc}},{\rm{F}}}$ gives the incentive to reduce *d*. Consequently, optimal values of *S* can be obtained at the maximum *l* but the optimal value of *d* depends on the charge transport properties characterized by the mobility lifetime product $\mu \tau$ of the utilized active material. In case of high quality poly-MAPbI_3_, the loss in ${\eta }_{{\rm{cc}},{\rm{F}}}$ for $d\le 100\,\mu {\rm{m}}$ is minimal and *d* can be chosen arbitrarily large within the investigated interval of $d\in \mathrm{[0.01}\,\mu {\rm{m}},100\,\mu {\rm{m}}]$ to ensure an optimal detector performance (see Fig. 4(a)). As opposed to this, the sensitivity in low quality poly-MAPbI_3_ is influenced by its poorer charge transport properties and an optimal value of *d* exists within the investigated interval that compromise the effect of effective filling (${\eta }_{{\rm{f}}}$) and charge collection (${\eta }_{{\rm{cc}},{\rm{F}}}$) (see Fig. 4(b)). However, the intrinsic property of the folded architecture enables to optimize the active layer thickness *d* without affecting the x-ray absorption efficiency and even in the low quality configuration high sensitivities up to $3\times {10}^{3}\,\mu {\rm{C}}/({{\rm{Gy}}}_{{\rm{air}}}{{\rm{cm}}}^{2})$ at $E=60\,{\rm{keV}}$ are feasible. Simulations of *S* at lower photon energies $E=20\,{\rm{keV}}$ and higher photon energies $E=500\,{\rm{keV}}$ (see Supplementary Fig. S1 and Fig. S2 for respective data) show qualitatively the same layout dependencies.

**Figure 5:**
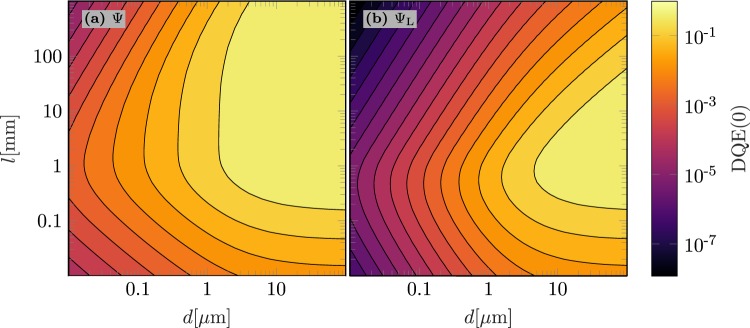
Simulated detective quantum efficiency DQE(0) of folded poly-MAPbI_3_ x-ray detectors in the high quality configuration ($\mu {\tau }_{{\rm{high}}}$) as a function of the active layer thickness *d* and the folding length *l* assuming a dark current density of ${J}_{{\rm{d}}}=3\times {10}^{-4}\,{\rm{mA}}/{{\rm{cm}}}^{2}$ (cf. ref.^30^). (**a**) Use ${\rm{\Psi }}$ and (**b**) use ${{\rm{\Psi }}}_{{\rm{L}}}$ as incoming photon flux. The x-ray energy is $E=60\,{\rm{keV}}$.

The detective quantum efficiency DQE(0) is a further important metric incorporating noise related influences on the performance of a detector. As the folded device architecture ensure high photon absorption efficiency also at higher energies to the disadvantage of an increased noise due to dark currents, Fig. 5 simulates the DQE(0) in folded poly-MAPbI_3_ detectors in case of typical ${\rm{\Psi }}$ and low incoming photon fluxes ${{\rm{\Psi }}}_{{\rm{L}}}$ assuming a constant dark current density of ${J}_{{\rm{d}}}=3\times {10}^{-4}\,{\rm{mA}}/{{\rm{cm}}}^{2}$ (cf. ref.^30^). Similar to the sensitivity analysis, calculations exemplarily use a photon energy of $E=60\,{\rm{keV}}$ and include different layout configurations (*l*, *d*).

At typical photon fluxes ${\rm{\Psi }}$ and large material conversion efficiencies ${\eta }_{{\rm{m}}}\gg 1$ as in case of MAPbI_3_, the detective quantum efficiency becomes approximately ${\rm{DQE}}(0)\approx {\eta }_{{\rm{f}}}{\eta }_{{\rm{x}}}$ even for large *l* and small *d* (see Eqs (19), (21) and (22)). Optimal DQE(0) values in folded device architectures can be reached if *d* and *l* are increased (see Fig. 5(a)) to enhance the effective fill factor ${\eta }_{{\rm{f}}}$ and the x-ray absorption efficiency ${\eta }_{{\rm{x}},{\rm{F}}}$ (see Eqs (11) and (14)). Thus, the DQE(0) analysis at typical fluxes results in similar design guidelines as the sensitivity simulations in Fig. 4(a). Only at larger *l* and smaller *d*, the influence of an increased total dark current per unit area plays a role as the DQE(0) decreases with increasing *l*.

According to Eq. (21), at lower fluxes ${{\rm{\Psi }}}_{{\rm{L}}}$, dark current contributions to the DQE(0) become more important resulting in an additional incentive to reduce *l* and raise *d* (see Eq. (22)). Consequently, an optimal parameter set $({l}^{\ast },{d}^{\ast })$ (see Fig. 5(b)) exists in the low flux case that provides a maximum DQE(0). As the sensitivity at this specific parameter set $S({l}^{\ast },{d}^{\ast })$ could only be optimized marginally (see sensitivity plateau in Fig. 4), $({l}^{\ast },{d}^{\ast })$ is seen as optimal parameter set for the folded device architecture.

### Performance evaluation and design rules for different x-ray applications

In order to evaluate the performance of folded device architectures with respect to different x-ray applications and to deduce respective design guidelines, Table 2 summarizes the simulated performance of folded poly-MAPbI_3_ devices based on low energies $E=20\,{\rm{keV}}$, mid energies $E=60\,{\rm{keV}}$, and high energies $E=500\,{\rm{keV}}$. To account for the complexity of fabricating high-quality poly-MAPbI_3_ layers both material quality configurations $\mu {\tau }_{{\rm{low}},{\rm{high}}}$ are considered. The performance is measured by the sensitivity *S* and the detective quantum efficiency DQE(0) in the typical ${\rm{\Psi }}$ and the low ${{\rm{\Psi }}}_{{\rm{L}}}$ flux case. Following the results of the sensitivity and DQE analysis, the optimal set of layout parameters $({l}^{\ast },{d}^{\ast })$ is determined by maximizing the ${\rm{DQE}}(0,{{\rm{\Psi }}}_{{\rm{L}}})$ at the respective energy *E*.

**Table 2:** Design guidelines of folded poly-MAPbI_3_ x-ray detectors in the high ($\mu {\tau }_{{\rm{high}}}$) and the low quality ($\mu {\tau }_{{\rm{low}}}$) configuration for different x-ray energies *E*.

*E*[keV]	*μτ*_high_					*μτ*_low_				
	l* [mm]	d* [*μ*m]	${\boldsymbol{S}}[\frac{{\boldsymbol{\mu }}{\bf{C}}}{{\bf{G}}{{\bf{y}}}_{{\bf{air}}}{\bf{c}}{{\bf{m}}}^{{\bf{2}}}}]$	DQE(0, Ψ_L_)	DQE(0)	l* [mm]	d* [*μ*m]	${\boldsymbol{S}}[\frac{{\boldsymbol{\mu }}{\bf{C}}}{{\bf{G}}{{\bf{y}}}_{{\bf{air}}}{\bf{c}}{{\bf{m}}}^{{\bf{2}}}}]$	DQE(0, Ψ_L_)	DQE(0)
20	0.25	100	297.4	0.93	0.96	0.17	75.9	130.6	0.78	0.92
60	1.66	100	4085	0.91	0.96	1.10	72.4	1830	0.75	0.91
500	83.2	100	3132	0.90	0.96	55.0	69.2	1429	0.70	0.90

The performance is measured by simulated sensitivities *S* and detective quantum efficiencies DQE(0) in the typical (Ψ) and the low (Ψ_L_) flux case. The optimal set of the folding length l* and the active layer thickness d* is determined by maximizing DQE(0, Ψ_L_). In the high quality configuration ($\mu {\tau }_{{\rm{high}}}$) the DQE(0) is limited by the signal loss due to the non effective filling ${\eta }_{{\rm{f}}} < 1$.

As the folded device architecture decouples absorption and charge collection, an efficient detection of photons with different energies can be ensured by adapting the folding length *l* to the respective photon energy *E* without affecting the charge collection of the detector. Consequently, folded detectors with active layer thicknesses of $d\le 100\,\mu {\rm{m}}$ enable high detector performances also for high energies as an efficient x-ray absorption can be ensured by rather thick ($l > 50\,{\rm{mm}}$) detector devices (see Table 2). Additionally, to a certain extent, the active layer thickness can be independently adjusted to the charge transport properties of the utilized conversion material. Thus, by slightly reduced active layers *d*, the folded architecture enables highly sensitive detectors even if a non optimal conversion material such as low quality poly-MAPbI_3_ is utilized.

**Figure 6:**
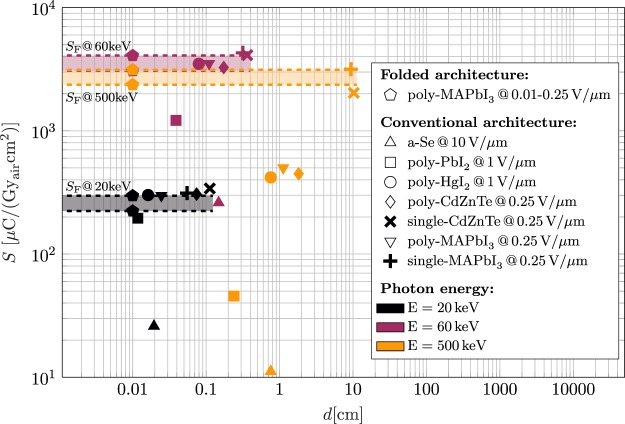
Simulated sensitivities *S*_F_ of folded poly-MAPbI_3_ x-ray detectors based on optimal layout parameters (see Table 2 and Supplementary Table S1) are compared to maximum achievable sensitivities *S* of conventional x-ray detectors based on state of the art conversion materials as a function of the active layer thickness *d*. Utilized photon energies are encoded with different colors and electric fields *F* are stated in the respective legend entry. In order to facilitate the comparison, *S*_F_ is additionally indicated with dashed lines.

Figure 6 finally compares sensitivities *S*_F_ of folded poly-MAPbI_3_ devices to maximum achievable sensitivities *S* of conventional x-ray detectors as a function of the active layer thickness *d*. Therefore, the sensitivity *S* of state of the art conversion materials in polycrystalline and single crystal states is simulated in the conventional planar detector design for various thicknesses *d*. In order to find maximum achievable values of *S* in the conventional layout, the optimal layer thickness *d* representing the optimal tradeoff between the x-ray absorption and the charge collection efficiency is determined. Sensitivities of folded poly-MAPbI_3_ detectors in Fig. 6 are based on the optimal detector layout in the high quality configuration determined in Table 2 and Supplementary Table S1. The different photon energies utilized in the simulations are encoded with different colors.

The electric fields *F* used in the simulations in Fig. 6 are stated in the respective legend entry. The specific field configuration needs to be carefully chosen as the electric field directly influences the charge collection efficiency ${\eta }_{{\rm{cc}}}$ as well as the dark current in the detector. In general, the level of an acceptable dark current density *J*_d_ is dependent on the specific detector application. Simulations in Fig. 6 based on a-Se, PbI_2_, HgI_2_, and CdZnTe utilize typical electric fields *F* reported in refs^14,59^ that correspond to maximum dark currents on the order of 10 pA/mm^2^ which is typically required in medical imaging. The relatively high level of dark currents in MAPbI_3_ conversion layers is a well-known challenge^29,30^ for this new class of materials. However, as the development of efficient perovskite conversion layers is just at the beginning, we believe that further research with respect to e.g. optimized charge-injection interfaces^30^ has the potential to drastically reduce dark current densities in MAPbI_3_. Thus, simulations of MAPbI_3_ devices utilize the minimum electric field used for the state of the art conversion materials. In order to conduct a realistic performance analysis, sensitivities in Fig. 6 of folded detectors based on p-MAPbI_3_ conversion layers utilize an ultra-low field configuration of *F* = 0.01 V/*μ*m as well (see Supplementary Table S1 for detailed design guidelines). At this field configuration a dark current level on the order of 100 pA/mm^2^ ^30^ can be achieved. We here note that according to Eq. (22) the relevant dark current density in the folded design is additionally dependent on the specific layout configuration (*l*, *d*).

Different to folded device architectures, the active layer thickness *d* needs to be adapted in the conventional layout in order to ensure an efficient photon absorption. At low energies $E=20\,{\rm{keV}}$ with the exception of a-Se, reachable sensitivities of folded and conventional detectors are comparable, independently whether polycrystalline or single crystal conversion materials are utilized and the required layer thicknesses in the conventional layout are in the practicable range of $d\in [100\,\mu {\rm{m}},750\,\mu {\rm{m}}]$ (polycrystalline) and $d\in [0.5\,{\rm{mm}},1.1\,{\rm{mm}}]$ (single crystals), respectively. For mid-energy photons with $E=60\,{\rm{keV}}$, folded detectors are able to compete with conventional devices based on poly-HgI_2_, poly-MAPbI_3_, poly-CdZnTe, single-MAPbI_3_, and single-CdZnTe. However, required layer thicknesses in the conventional design exceed *d* = 1 mm which is feasible in case of single crystals but potentially requires more complex and time consuming processing if polycrystalline materials are utilized (cf. poly-CdZnTe deposition rate of 25 nm/s in ref.^24^). Furthermore, folded devices profit from much smaller required applied voltages *U* = *Fd* decreasing the risk for the read out electronics. With an electric field of *F* = 0.25 V/*μ*m and an active layer thickness of *d* = 100 *μ*m, the required applied voltage in the folded poly-MAPbI_3_ layout is *U* = 25 V whereas e.g. single-CdZnTe requires *U* = 281 V@20 keV and *U* = 908 V@60 keV to achieve the same sensitivity. In case of high energies *E* = 500 keV, charge transport properties of state of the art polycrystalline materials at typically values of *F* are not sufficient to reach the sensitivity of folded device architectures. Although, the sensitivities of conventional detectors with polycrystalline conversion materials could be improved by utilizing higher values of *F*, an increase of the electric field is not preferable as this would increase the noise due to dark current. In principle, due to their optimal charge transport properties, single crystal CdZnTe and MAPbI_3_ would enable conventional detectors with equally high sensitivities as reachable with folded detectors. However, here the required single crystal layer thickness is in the range of 10 cm. Challenges in growing high quality single crystals in such large dimensions, extremely high required voltages ($U\approx 25\,{\rm{kV}}$), and decreased device performance (e.g. degraded spatial, temporal and energy resolution caused by the thick active layer) make the realization of such detectors highly unlikely. As opposed to this, we believe that challenges in the fabrication of folded devices such as the structured deposition of the conversion layers and the detector read out are solvable. The former could, e.g., be realized by making use of inkjet-printing^60^ for a patterned deposition enabling a folding of the detector foil. With respect to the latter challenge, already developed application specific integrated circuits based on either energy integrating or hybrid pixel technologies can be connected to the folded detector through the bottom of every fold (see Fig. 2). Finally, once the fabrication of a folded device is successfully established, the adaption of the folding length *l* to meet the requirements of a specific application is feasible making the folded detector layout an interesting alternative for different x-ray applications. Competing with the high performance of single crystals with respect to the detective quantum efficiency is more challenging. Even if a relatively high dark current density of ${J}_{{\rm{d}}}={10}^{-3}\,{\rm{mA}}/{{\rm{cm}}}^{2}$ is assumed, the linear system model applied here predicts values of ${\rm{DQE}}(0,{{\rm{\Psi }}}_{{\rm{L}}})\approx 0.95\,@\,20\,{\rm{keV}}$, ${\rm{DQE}}(0,{{\rm{\Psi }}}_{{\rm{L}}})\approx 0.99\,@\,60\,{\rm{keV}}$, ${\rm{DQE}}(0,{{\rm{\Psi }}}_{{\rm{L}}})\approx 0.99\,@\,500\,{\rm{keV}}$ in case of conventional detectors based on single-MAPbI_3_ and single-CdZnTe. Thus, referring to the DQE(0), the competitiveness of the folded detector design is dependent on the material quality of the poly-MAPbI_3_ conversion layer (see Table 2 and Supplementary Table S1 for the respective DQE values of the folded design).

Summarizing, in all three energy configurations, the sensitivity of folded poly-MAPbI_3_ x-ray detectors is comparable to the sensitivities of high quality single crystal CdZnTe and MAPbI_3_. Even if low quality poly-MAPbI_3_ is assumed, the folded design enables a considerably high performance with values of *S* in the same order of magnitude as single-CdZnTe and single-MAPbI_3_. With respect to the detective quantum efficiency and especially in case of low incoming photon fluxes, the influence of the poly-MAPbI_3_ material quality is higher. Although folded devices based on high quality poly-MAPbI_3_ show considerably high performance, DQE(0) values are slightly reduced compared to planar detectors based on high quality single crystal CdZnTe and MAPbI_3_ (see Table 2 and Fig. 6).

We here note that the folded device architecture might also enable ultra high spatial resolution 1D detectors as single folds can define pixels whose width is only dependent on the thickness of the substrate foil and the conversion material sandwiched between the electrodes (see Fig. 2). Furthermore, as single folds are separated by the substrate, folded devices avoid the problem of charge sharing effects due to carrier diffusion and coulomb repulsion and potentially improve both the spatial as well as the energy resolution. However, in this respect it is important to note, that even with physically separated pixels, the folded design is not able to avoid K-fluorescence re-absorption within neighboring pixels. Typical path lengths *λ* (attenuations lengths) of the fluorescence photons of lead (Pb) and iodine (I) in MAPbI_3_ can be calculated by the energy *E*_K_ of the K-edges (${E}_{{\rm{K}},{\rm{Pb}}}=88\,{\rm{keV}}$, ${E}_{{\rm{K}},{\rm{I}}}=33\,{\rm{keV}}$^61,62^) and the mass attenuation coefficients *α*/$\rho$ in MAPbI_3_^57^ already used for the simulations. Attenuation lengths of ${\lambda }_{{\rm{K}},{\rm{Pb}}}=1\,{\rm{mm}}$ and ${\lambda }_{{\rm{K}},{\rm{I}}}=193\,\mu {\rm{m}}$ in MAPbI_3_ indicate that both the spatial as well as the energy resolution of folded detectors especially at higher energies can be affected by K-fluorescence re-absorption. However, the exact influence of K-fluorescence re-absorption and moreover the influence of secondary electron path lengths within the conversion material and Compton scattering within the specimen and the conversion material on the spatial and energy resolution needs to be further investigated.

## Conclusion

In summary, in this article, we have proposed a folded detector layout that enables to decouple the x-ray absorption and the charge collection. The sensitivity *S* of folded poly-MAPbI_3_ x-ray detectors was investigated for the case of polycrystalline layers with high and low material quality. Folded detectors allow for high sensitivities even in case of low quality poly-MAPbI_3_ conversion layers. Furthermore, we have presented an analysis of the detective quantum efficiency of folded poly-MAPbI_3_ devices at typical and low incoming photon fluxes that shows additional limitations for the folding length. Based on the analysis of the sensitivity and the detective quantum efficiency, the optimal layout parameter set $({l}^{\ast },{d}^{\ast })$ was determined for high and low quality poly-MAPbI_3_ layers and their performance was evaluated for different photon energies. The resulting design guidelines underline the inherent benefit of the folded detector layout as the folding length *l* and the active layer thickness *d* can be optimized independently within technological limits. Consequently, high detector performance can be achieved even in case of low quality poly-MAPbI_3_ as the x-ray absorption efficiency can be adapted to the utilized photon energy and the active layer thickness can be optimized with respect to the charge transport properties of the conversion material. We have finally shown that folded devices are a promising pathway to combine the superior detector performance typically found for single crystals with the processing and cost advantages of polycrystalline layers.

## Supplementary information

Supplementary

## Data Availability

The datasets generated and analysed during the current study are available from the corresponding author on reasonable request.
