# Supplementary material for: Simulation and design of folded perovskite x-ray detectors
Source: Sci Rep. 2019 Mar 26;9:5231. doi: 10.1038/s41598-019-41440-6 (PMC6435812; doi:10.1038/s41598-019-41440-6)
Supplement: Supplementary file 1 — Supplementary [file 41598_2019_41440_MOESM1_ESM.pdf]

Simulation and design of folded perovskite x-ray detectors

Henning Mescher[1,2], **Elias Hamann**[3], **and Uli Lemmer**[1,2,*]

[1]Light Technology Institute, Karlsruhe Institute of Technology (KIT), 76131 Karlsruhe, Germany
[2]Institute of Microstructure Technology, Karlsruhe Institute of Technology (KIT), 76344 Eggenstein-Leopoldshafen, Germany
[3]Institute for Photon Science and Synchrotron Radiation, Karlsruhe Institute of Technology (KIT), 76344 Eggenstein-Leopoldshafen, Germany
[*]uli.lemmer@kit.edu

ABSTRACT

A variety of medical, industrial, and scientific applications requires highly sensitive and cost-effective x-ray detectors for photon energies ranging from keV to MeV. Adapting the thickness of polycrystalline or single crystal conversion layers especially to high-energy applications increases the complexity of fabrication and potentially decreases the performance of conventional direct conversion x-ray detectors. To tackle the challenges with respect to the active layer thickness and to combine the superior performance of single crystal materials with the low-cost nature of polycrystalline conversion layers, we investigate thin film x-ray detector technologies based on a folded device architecture. Analytical models simulating the sensitivity and the detective quantum efficiency (DQE) are used to evaluate the performance of folded detectors based on polycrystalline organic-inorganic perovskite semiconductors in various layout configurations and for different photon energies. Simulations of folded perovskite devices show high sensitivities. The DQE analysis introduces additional noise related boundary conditions for the folding length. A comparison with conventional detectors based on state of the art conversion materials at different photon energies demonstrates the potential of the folded detector layout as simulated sensitivities are comparable to single crystal detectors.

Supplementary Information

Simulated sensitivities at lower photon energies

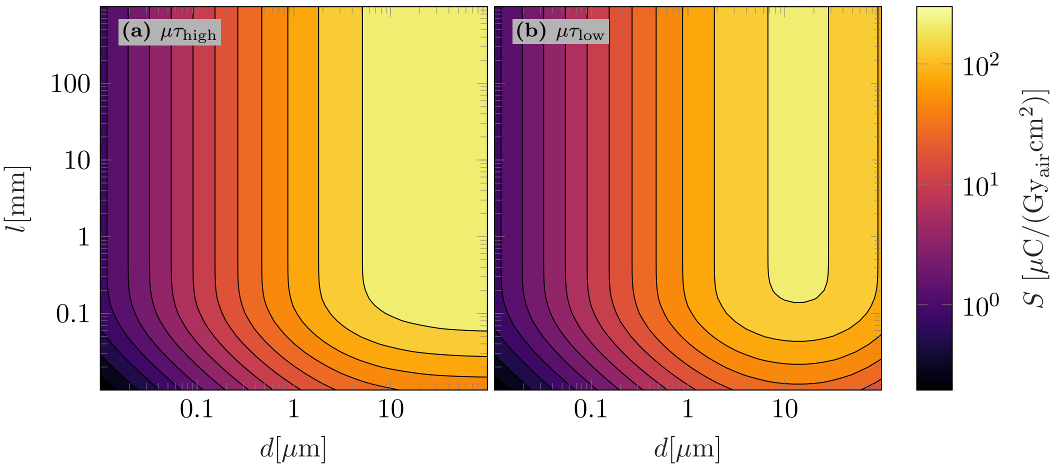

Figure 1. Simulated sensitivities S of folded poly-$MAPbI_3$ x-ray detectors as a function of the active layer thickness d and the folding length l. (a) use $\mu\tau_{\mathrm{high}}$ and (b) use $\mu\tau_{\mathrm{low}}$ to simulate S in the high and the low quality configuration. The x-ray energy is $E = 20\,\mathrm{keV}$.

Simulated sensitivities at higher photon energies

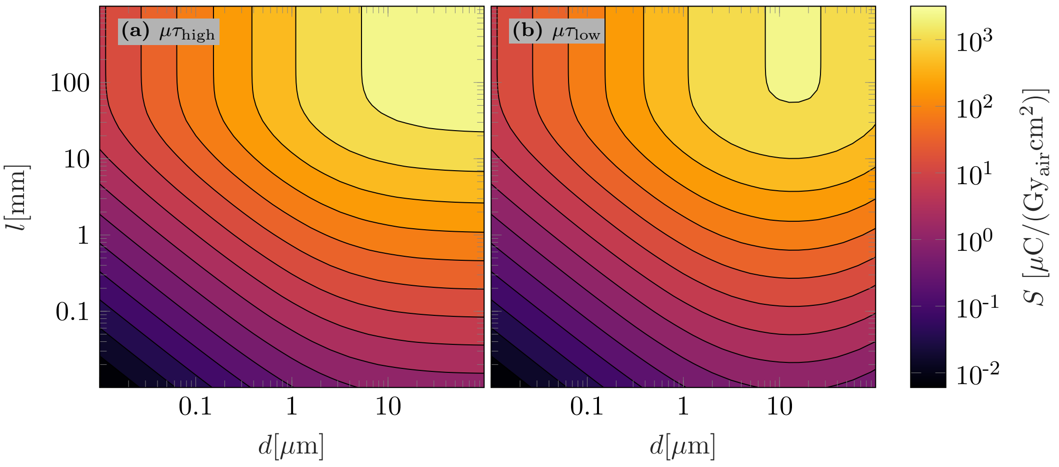

Figure 2. Simulated sensitivities S of folded poly-$MAPbI_3$ x-ray detectors as a function of the active layer thickness d and the folding length l. (a) use $\mu\tau_{\mathrm{high}}$ and (b) use $\mu\tau_{\mathrm{low}}$ to simulate S in the high and the low quality configuration. The x-ray energy is $E = 500\,\mathrm{keV}$.

Design guidelines of folded poly-$MAPbI_3$ detectors at a lower electric field

Table 1. Design guidelines of folded poly-$MAPbI_3$ x-ray detectors in the high ($\mu\tau_{high}$) and the low quality ($\mu\tau_{low}$) configuration for different x-ray energies E and an electric field $F = 0.01\,V/\mu m$. The performance is measured by simulated sensitivities S and detective quantum efficiencies DQE(0) in the typical (Ψ) and the low (Ψ_L) flux case. The optimal set of the folding length l^* and the active layer thickness d^* is determined by maximizing DQE(0,Ψ_L). In the high quality configuration ($\mu\tau_{high}$) the DQE(0) is limited by the signal loss due to the non effective filling $\eta_f < 1$.

	$\mu\tau_{high}$					$\mu\tau_{low}$				
E[keV]	l^*[mm]	d^*[μm]	$S[\frac{\mu C}{Gy_{air}cm^2}]$	DQE(0,Ψ_L)	DQE(0)	l^*[mm]	d^*[μm]	$S[\frac{\mu C}{Gy_{air}cm^2}]$	DQE(0,Ψ_L)	DQE(0)
20	0.40	100	223.5	0.97	0.97	0.14	12.0	36.21	0.58	0.76
60	2.69	100	3077	0.97	0.97	0.95	11.0	526.1	0.55	0.74
500	138	100	2367	0.97	0.97	49.0	9.55	440.6	0.50	0.71
